# Correlation of the FIB-4 Liver Biomarker Score with the Severity of Heart Failure

**DOI:** 10.3390/medicina60121943

**Published:** 2024-11-26

**Authors:** Roxana Buzas, Paul Ciubotaru, Alexandra Corina Faur, Marius Preda, Melania Ardelean, Doina Georgescu, Patrick Dumitrescu, Daniel Florin Lighezan, Mihaela-Diana Popa

**Affiliations:** 1 1st Medical Semiology, Internal Medicine, Department V, “Victor Babes” University of Medicine and Pharmacy, 300041 Timisoara, Romaniapaul.ciubotaru@umft.ro (P.C.); georgescu.doina@umft.ro (D.G.);; 2 Center for Advanced Research in Cardiovascular Pathology and in Hemostaseology, 300041 Timisoara, Romania; 3 Department of Anatomy and Embryology, “Victor Babeș” University of Medicine and Pharmacy Timișoara, Eftimie Murgu Square, No. 2, 300041 Timișoara, Romania; 4 Second Discipline of Surgical Semiology, Department IX—Surgery—1, “Victor Babes University of Medicine and Pharmacy, 300041 Timisoara, Romania; 5 Second Clinic of General Surgery and Surgical Oncology, Emergency Clinical Municipal Hospital, 300079 Timisoara, Romania; 6 Breast Surgery Research Center, “Victor Babes” University of Medicine and Pharmacy, 300041 Timisoara, Romania; 7 General Medicine, “Victor Babeș” University of Medicine and Pharmacy from Timișoara, Eftimie Murgu Sq. No. 2, 300041 Timișoara, Romania; patrick.dumitrescu@student.umft.ro; 8 Department of Microbiology, “Victor Babes” University of Medicine and Pharmacy, 300041 Timisoara, Romania

**Keywords:** heart failure, liver cirrhosis, liver fibrosis, LVEF, FIB-4 score

## Abstract

*Background and Objectives*: Heart failure is associated with high morbidity and mortality and linked with several pre-existing health conditions and risk factors. Early detection and prompt management in heart failure improves patient outcomes. Liver involvement is associated with heart failure disease progression, and hence liver biomarkers and liver fibrosis may have a prognostic impact. Several blood test based markers and scoring systems estimate liver fibrosis and hence can be useful prognostic tools. *Materials and Methods*: We retrospectively analyzed a series of 303 patients with decompensated heart failure in a city in western Romania over a period of 6 months. Several biochemical parameters were measured, the FIB-4 score was estimated and echocardiography was performed. Results for targeted variables are presented using descriptive statistics. Patients were analyzed based on their LVEF categories. Statistical analysis was based on ANOVA one-way tests for continuous variables and Chi-square tests for categorical variables. Pairwise comparisons were performed based on Bonferroni adjusted significance tests. The correlations between FIB-4 score, LVEF and NT-pro BNP in patients with and without diabetes and hypertension were explored using Spearman’s correlation coefficient. *Result*: Age, gender, NYHA class, death, history of (h/o) type 2 diabetes mellitus (T2DM), h/o coronary artery disease (CAD), h/o arrhythmias, sodium, potassium, creatinine, eGFR, uric acid, NT-pro BNP, left atrial volume, LDL, HDL, and TG were analyzed by LVEF categories using ANOVA one-way tests, Chi-square tests, and Bonferroni correction comparisons. We found a strong statistically significant correlation between each of NT-pro BNP, left atrial volume, LDL, and HDL with the LVEF categories. *Discussion*: Early detection of cardiac dysfunction leads to better management in patients with cardiovascular risk factors including diabetes and hypertension. High LDL and low HDL levels contribute to a reduction in left ventricular (LV) function. Available literature suggests the FIB-4 score as superior to other non-invasive markers of fibrosis. It utilizes the patient’s age, platelet count, AST, and ALT, which can be available retrospectively, making it an easy and inexpensive tool. FIB-4 score has a few limitations. *Conclusions*: Our study has shown a statistically significant positive correlation between severity categories of LVEF and FIB-4 score for heart failure patients with and without diabetes, and for heart failure patients with or without hypertension. We propose the implementation of FIB-4 score as a prognostic tool for heart failure.

## 1. Introduction

Heart failure is a complex multi-organ involvement disease associated with high morbidity and mortality. Heart failure has a high incidence in the elderly population, of around 10 per 1000 in people over 65, across the globe [1]. According to the 2021 ESC guidelines [2], the incidence of heart failure in Europe is about 3 to 5/1000 person–years in adults [3,4] whereas the prevalence of HF appears to be 1 to 2% of adults [5,6]. According to the 2022 AHA/ACC/HFSA Guideline [7] for the Management of Heart Failure, total deaths caused by heart failure have increased from 275,000 in 2009 to 310,000 in 2014. The symptoms and signs of heart failure are developed due to structural and/or functional dysfunction of the heart. Left heart failure occurs when the left ventricle fails to pump blood efficiently, leading to fluid accumulation in the lungs and other organs. Left heart failure is caused as a result of ischemic heart disease, myocardial infarction, systemic hypertension, and valvulopathy including mitral regurgitation, aortic stenosis, aortic regurgitation and dilated cardiomyopathy [8]. Right heart failure occurs as a result of conditions including but not limited to acute respiratory distress, pulmonary embolism, right ventricular myocardial infarction, right heart valvulopathy, cardiomyopathy, constrictive pericarditis, cardiac tamponade, congenital heart disease, and pulmonary hypertension, as well as due to left heart failure [9,10].

Furthermore, health conditions such as diabetes mellitus, obesity, kidney disease, anemia, sleep apnea, myocarditis, endocarditis, and inflammatory conditions may also lead to heart failure [11,12]. While sometimes as a marker there can be the presence of arrhythmias with potential to produce acute decompensation of cardiac function [13], for many of these risk factors, paradoxical effects have also been described in terms of risk or protection against the occurrence of cardiovascular diseases. Some risk factors for heart failure include genetic predisposition, a sedentary lifestyle, age, smoking, dyslipidemia and hyperhomocysteinemia [11,13]. Heart failure can be classified into heart failure with preserved ejection fraction (HFpEF), with an ejection fraction above 50 percent, heart failure with mid-range ejection fraction (HFmrEF), with an ejection fraction from 41 to 49 percent, and heart failure with reduced ejection fraction (HFrEF), with an ejection fraction below 40 percent. Early detection and prompt management are crucial in heart failure to improve patient outcomes.

Right heart failure can lead to hepatic congestion and liver injury. The degree of liver injury will depend upon the magnitude of congestion or impaired perfusion [14]. It occurs due to high pressure transmitted from the right ventricle to the hepatic veins and elevated central venous pressure (CVP) [15]. The resultant decrease in hepatic arterial blood flow leads to congestive hepatopathy, cardiac cirrhosis, and liver fibrosis. This is the reason for elevated liver enzymes and jaundice that may be found in acute heart failure cases. Patients may complain of right upper quadrant pain due to ascites and a strained liver capsule [16]. The presence of hepatojugular reflux can differentiate between primary liver disease and cardiac cirrhosis, since it may not be as pronounced in the latter case.

Studies have shown that the involvement of the liver has an important role in heart failure. This is so because the advanced stages of heart failure may be associated with acceleration of disease progression [17]. Furthermore, diabetes and heart failure are strongly interrelated [18,19]. After COVID-19 viral infection, patients have demonstrated cardiac rhythm disturbances, myositis, myocarditis, and conjunctivitis, and this has further contributed to the morbidity and mortality of hospitalized patients with heart failure and liver cirrhosis [20,21,22,23]. COVID-19 infection is known to involve the pancreas [24,25,26] and worsen patients’ insulin resistance [27,28], further strengthening the link between liver fibrosis, insulin resistance and heart failure. This suggests a need to develop prognostic scores based on liver biomarkers in heart failure patients with or without diabetes mellitus.

Liver fibrosis and liver stiffness measurement (LSM) have prognostic value on long-term outcome and mortality [29,30,31,32] and they are measured by gold standard liver biopsy and non-invasive elastography [33,34].

Blood test-based markers and scoring systems can also be used to estimate liver fibrosis. Brain natriuretic peptide (BNP) or NT-pro BNP can differentiate acute heart failure from other causes of shortness of breath. Troponin levels can determine acute coronary syndrome, i.e., acute myocardial infarction. Bilirubin level increases in liver injury due to volume overload and serves as an independent predictor of the severity of heart failure in terms of reduction of ejection fraction [35]. In the setting of acute heart failure, type III pro-collagen peptide level may serve as a biomarker for liver involvement [36]. However, some liver tests can be influenced by the administration of active cardiovascular medications. Digoxin, one of the drugs used in the treatment of patients with heart failure and atrial fibrillation in order to obtain better control of the heart rate [37], seems to have an anti-inflammatory role at the liver level. It protects the liver against the occurrence of alcoholic or non-alcoholic hepatic steatosis [38] although, when administered to patients with liver cirrhosis, it is associated with a negative prognosis [39]. Peripheral brain-derived neurotrophic factor (BDNF) levels are found to be reduced in patients with heart failure with reduced ejection fraction (HFrEF) and may be of some prognostic value [40]. However, BDNF measurements are not yet popular, due to several challenges. Plasma BDNF levels are strongly influenced by platelet levels, hence the absence or presence of liver disease, as well as daily use of anti-platelet therapy, especially clopidogrel, are significant confounding factors for BDNF levels [41]. Decreased peripheral BDNF levels are found in depression and in some neurodegenerative conditions [42,43], which is another confounding factor.

A few prognostic scores based on liver biomarkers are available [44], such as the Model for End-stage Liver Disease excluding INR (MELD-XI), non-alcoholic fatty liver disease (NAFLD) fibrosis score (NFS), fibrosis 4 (FIB-4) score fibrosis 5 (FIB-5) score, and the triglyceride and glucose (TyG) index score. The fibrosis 4 (FIB-4) score is a non-invasive tool used to assess liver fibrosis. It is calculated using a formula that takes into account age, platelet count, and the liver enzymes AST and ALT [45,46]. The FIB-4 score helps estimate the level of scarring in the liver, which is crucial for diagnosing and monitoring liver disease, wherein a lower score indicates a lesser degree of fibrosis [47]. An FIB-4 score < 1.45, having negative predictive value of 90% for advanced fibrosis, is considered as low risk, whereas an FIB-4 score > 3.25, having 97% specificity and 65% positive predictive value for advanced fibrosis, is considered as high risk [45]. To make use of the FIB-4 score in predicting the severity of heart failure, it is appropriate to rule out certain conditions that can contribute to fibrosis, such as the viral infections hepatitis B virus (HBV) and hepatitis C virus (HCV), alcoholic liver disease, and hemochromatosis, among others [48]. In addition to the FIB-4 score, the fibrosis 5 (FIB-5) score is another non-invasive index used to assess liver fibrosis. Along with the four parameters that are included in the FIB-4 score, it incorporates an additional variable, the aspartate aminotransferase-to-platelet ratio index (APRI) [49]. Interpretation of the FIB-5 score are low risk (FIB-5 < 1.25), intermediate risk (1.25 ≤ FIB-5 < 2.5), and high risk (FIB-5 ≥ 2.5). However, it is essential to note that FIB-5 score is not as widely validated or adopted as FIB-4 score [49]. Hence we excluded it from our study at this point. Furthermore, diet plays an important role [50]. The triglyceride and glucose (TyG) index score is another similar non-invasive tool that is described as a biomarker for insulin resistance and assesses liver fibrosis severity by estimating formula-based liver stiffness, which involves parameters including ALT, AST, BMI, platelet count, and presence or absence of diabetes [51,52] and may predict mortality in heart failure patients [53,54]. However studies have shown its lack of accuracy in detecting early fibrosis, and have failed to support the association between the TyG index and cardiovascular prognosis [55,56]. Hence we excluded it from our study.

## 2. Methods

We aimed to evaluate liver biomarkers, using the FIB-4 score as a measure of evaluation of the severity of heart failure. We retrospectively analyzed a series of 303 consecutive patients with known heart failure admitted to the Internal Medicine Department of the Municipal County Emergency University Hospital, Timisoara, between 1 January 2023 to 31 December 2023. All patients either presented themselves or were transported by ambulance to the ER, where they were evaluated and admitted to the Internal Medicine department with decompensated heart failure.

During their hospital stay, all patients underwent echocardiography, performed by an experienced cardiologist. Planimetric ejection fractions were measured using a General Electric GE Vivid E9 Ultrasound System. Patients were divided into three groups based on their ejection fraction: the first group comprised patients with preserved ejection fraction (HFpEF), the second group comprised patients with mildly reduced or mid-range ejection fraction (HFmrEF), and the third group comprised patients with reduced ejection fraction (HFrEF) according to the European Society of Cardiology (ESC) 2023 guidelines.

Blood samples were taken to measure several biochemical parameters: aspartate transaminase, alanine aminotransferase, bilirubin, albumin, alkaline phosphatase and platelet count among others. NT-pro BNP levels were also measured for all patients during the first 24 h after admission, from blood samples, using a MINI VIDAS^®^ (bioMérieux, Marcy-l'Étoile, France) compact multiparametric immune analyzer, with results expressed in pg/mL.

Exclusion criteria were the chronic viral infections HBV and HCV, patients with an acknowledged history of heavy alcohol consumption, storage diseases (hemochromatosis) or autoimmune liver diseases and severe steatohepatitis, solid organ neoplasms, and malignant or chronic hematological diseases. In addition, patients that were intubated or were resuscitated after sudden cardiac arrest in the ER were not included in our analysis.

The FIB-4 score was estimated for all patients, with age in years, ALT and AST in IU/L, and platelet count in 10 raised to 9/L: FIB-4 score = (age × AST)/(platelet count × √ALT); this provided values from 0.2 to 10 [46].

Results for targeted variables were presented using descriptive statistics (mean, standard deviation, range, median, interquartile range) for continuous data, and counts with associated percentages for categorical data.

We analyzed our patients based on the following three left ventricular ejection fraction (LVEF) categories: LVEF > 50%, LVEF 49–40%, LVEF < 40%. Statistical analysis was based on ANOVA one-way tests for continuous variables and Chi-square tests for categorical variables, with the corresponding *p*-values presented in the summary tables. Pairwise comparisons were performed based on Bonferroni adjusted significance tests, with the corresponding significance (S; *p* < 0.05) or no significance (NS; *p* > 0.05) being reported. The *p*-values based on Bonferroni correction comparison for comparing the LVEF > 50% category with the LVEF 49–40% category patients are flagged under *p*-value 1 column in the summary tables, while *p*-value 2 flags the comparison results of the LVEF > 50% category with the LVEF < 40% category patients and *p*-value 3 flags the comparison results of the LVEF 49–40% category with the LVEF < 40% category patients.

The study was approved by the Scientific Research Ethics Committee of the Municipal County Emergency University Hospital, Timisoara, Romania (approval number: E-2407/15.04.2024), and was conducted in accordance with the Helsinki Declaration. Written informed consent was obtained as a part of a routine procedure from all the patients admitted at our university hospitals for further research publication and educational purposes.

## 3. Result

The following tables show the results of this study.

Table 1 shows the summary of main characteristics by LVEF categories for the patients included in our statistical analysis. The *p*-values are obtained by ANOVA one-way tests and Chi-square tests. *p*-values 1, 2, 3 are based on Bonferroni correction comparisons, indicating S for significant *p*-values and NS for non-significant *p*-values. Continuous data are summarized as mean (standard deviation); minimum and maximum value; median and associated quartiles (Q1–25 percentage quartile; Q3–75 percentage quartile). Quartiles are obtained with Tukey’s method. Categorical data are presented as counts (percentages).

Table 2 gives a summary of co-morbidities by LVEF categories for the patients included in our statistical analysis. The *p*-values are obtained with Chi-square tests. The *p*-values 1, 2, 3 are based on Bonferroni correction comparisons, indicating S for significant *p*-values and NS for non-significant *p*-values. Categorical data are presented as counts (percentages).

Table 3 gives a summary of main laboratory test results by LVEF categories for the patients included in our statistical analysis. The *p*-values are obtained with ANOVA one-way tests. The *p*-values 1, 2, 3 are based on Bonferroni correction comparisons, indicating S for significant *p*-values and NS for non-significant *p*-values. Continuous data are summarized as mean (standard deviation); minimum and maximum value; median and associated quartiles (Q1–25 percentage quartile; Q3–75 percentage quartile). Quartiles are obtained with Tukey’s method.

Table 4 summarizes the correlation between FIB-4 score and LVEF and NT-pro BNP in patients with and without diabetes and hypertension.

### 3.1. Interpretation of Correlations with/Without Diabetes

A weak positive (i.e., direct) correlation between LVEF and FIB-4 score was revealed using Spearman’s correlation coefficient (r = 0.378, *p* < 0.001) for patients with diabetes. Similarly, for patients without diabetes, a weak positive correlation between LVEF and FIB-4 score was obtained (r = 0.344, *p* < 0.001).

A very weak positive non-significant correlation between NT-pro BNP and FIB-4 score was revealed using Spearman’s correlation coefficient (r = 0.045, *p* = 0.621) for patients with diabetes. Similarly, for patients without diabetes, a very weak positive non-significant correlation between NT-pro BNP and FIB-4 score was obtained (r = 0.001, *p* = 0.993).

### 3.2. Interpretation of Correlations with/Without Hypertension

A weak positive correlation between LVEF and FIB-4 score was revealed using Spearman’s correlation coefficient (r = 0.355, *p* < 0.001) for patients with hypertension. However, for patients without hypertension, a moderate positive correlation between LVEF and FIB-4 score was obtained (r = 0.506, *p* = 0.003).

A very weak positive non-significant correlation between NT-pro BNP and FIB-4 score was revealed using Spearman’s correlation coefficient (r = 0.002, *p* = 0.979) for patients with hypertension. Similarly, for patients without hypertension, a very weak negative non-significant correlation between NT-pro BNP and FIB-4 score was obtained (r = −0.100, *p* = 0.580).

## 4. Discussion

Diabetes and hypertension are very highly prevalent co-morbidities in the general population and are well-known risk factors for acute coronary syndrome and heart failure [57]. Our study has shown a statistically significant positive (i.e., direct) correlation between LVEF and FIB-4 scores for patients with diabetes as well as without diabetes and for patients with and without hypertension. Studies have shown subclinical left ventricular dysfunction in asymptomatic diabetic patients, which is further linked with the duration of diabetes [58]. Hence early detection of diabetic heart disease seems crucial in preventing the future development of heart failure. Similarly, there are reports of reduction of basal longitudinal strain, altered left ventricular geometry and left ventricular dysfunction in patients with hypertension [59]. Early detection of cardiac dysfunction may help in managing patients who have several cardiovascular risk factors including diabetes and hypertension.

We found a strong statistically significant correlation between each NT-pro BNP, left atrial volume, LDL, and HDL with the LVEF categories. NT-pro BNP, high sensitivity C-reactive protein (hs-CRP), and cardiac troponin I are known for their potential as predictive biomarkers of LV remodeling [60]. Cardiovascular disease is a chronic atherosclerosis-driven pathophysiological phenomenon [61]. Dyslipidemia in terms of high LDL and low HDL levels may contribute to reduced LV function by decreasing myocardial flow reserve and capillary density, and by increasing capillary endothelial cell apoptosis following ischemia and reperfusion [62,63]. Some studies that have shown independent association between low HDL levels and subclinical left ventricular systolic dysfunction [64], as well as statistically significant inverse correlations between LDL levels, NT-pro BNP levels and LVEF [65]. Dyslipidemia has an effect on progressive LV dilation and management of dyslipidemia may decrease microvascular perfusion [66].

Available literature suggests that FIB-4 is an accurate and cost-effective method for the prediction of liver fibrosis, which may reduce the need for liver biopsy [67]. The FIB-4 score was considered as superior to other noninvasive markers of fibrosis in patients with NAFLD [68], with a high negative predictive value for excluding advanced fibrosis [69]. This score utilizes the age factor, platelet count, AST and ALT. Age and platelet count have demonstrated strong correlations with advanced fibrosis [70,71]. Raised levels of AST and ALT have also been associated with advanced fibrosis [72,73,74]. Hence, a composite scoring system involving these parameters, such as the FIB-4 score, provides an easy assessment of liver fibrosis without any invasive procedures and even without any additional expensive blood tests. In addition, it can be applied to retrospective data. Moreover, we propose routine use of the FIB-4 score as a prognostic tool for heart failure, which utilizes age (in years), platelet count (in 10 raised to 9/L), AST (in IU/L) and ALT (in IU/L) for its calculation, all of which are feasible to inexpensively collect and calculate for primary care physicians.

Predicting secondary events after initial events of heart failure poses a challenge. Anstee et al. [75] have investigated the association between FIB-4 and subsequent liver events, cardiovascular events, and all-cause mortality in more than 44,000 individuals with obesity and/or type 2 diabetes and concluded that sequential measurement of FIB-4 score provides a pragmatic risk assessment system for liver-related events (LREs), major adverse cardiovascular events (MACEs), and also mortality. Kamada et al. [76] found that the FIB-4 index is a reliable non-invasive tool to predict not only LREs but also extrahepatic events like cancers and MACEs. Such studies suggest that FIB-4 score could also be used as a predictive biomarker for secondary prevention.

However, the FIB-4 score is not without limitations. It was developed in a cohort of subjects that did not include patients <35 or >65 years old, and the score has been shown to be less reliable in these patients, i.e., the very young and the very elderly groups [77]. The score’s validity in those age groups needs to be tested, given the age in the numerator of the score. Importantly, the AST in the numerator may overestimate fibrosis in those patients who use alcohol, since one cannot clearly distinguish (and hence exclude) a patient’s high vs. moderate vs. mild alcohol use over a long span in their history. Finally, there are no data available on the use of FIB-4 score in patients treated with direct-acting antivirals and achieved sustained virologic response. In addition, since AST and ALT may normalize with sustained virologic response, the FIB-4 score may be lower unless the patient has underlying non-alcoholic fatty liver disease (NAFLD) and/or very low platelets. Therefore, this is one cohort of patients who should be tested with this score; however, it is unrelated to heart failure. The pathophysiology of liver function abnormalities in HF patients is a combination of both congestion (increased CVP) and reduced cardiac output. While investigating the relationship of HF with the severity of TR, Lau et al. [78] studied data of patients admitted to the cardiology unit of a tertiary hospital with a diagnosis of left, right, or congestive HF, and found that the elevation of the cholestatic profile was significantly associated with the degree of tricuspid incompetence, i.e., patients with moderate or severe TR had significantly greater ALP, GGT, and bilirubin than those with no or mild TR, but not ALT or AST.

Cao et al. [79] suggested that liver fibrosis scores (LFSs) are useful tools for identifying and re-stratifying patients with HfpEF, through a comparative study among different LFSs. In their analysis, the NFS appears to have superior predictive and prognostic utility compared to several other scores including FIB-4. This calls for a similar comparative study among several non-invasive LFSs from a larger data set. Our overall findings and limitations call for more studies across different centers that can use this simple score and produce data that can further validate this score.

## 5. Conclusions

Our data establish a statistically significant direct relationship between LVEF severity categories and the FIB-4 score for patients with or without diabetes, and for heart failure patients with or without hypertension. We promote the application of LFSs as a prognostic tool for heart failure patients, and propose the implementation of the FIB-4 score as one such simple, non-invasive, non-expensive, LFS.

## Figures and Tables

**Table 1:** Main characteristics by LVEF categories.

	LVEF > 50% (*n* = 198)	LVEF 49–40% (*n* = 54)	LVEF < 40% (*n* = 51)	*p*-Value	*p*-Value 1	*p*-Value 2	*p*-Value 3
Age (years)							
Mean (SD)	75.35 (9.385)	74.31 (10.731)	70.04 (12.625)	0.005	NS	S	NS
Min; Max	47; 96	46; 96	47; 94				
Median (Q1; Q3)	76.0 (69.0; 83.0)	77.0 (67.0; 82.0)	70.0 (60.5; 80.0)				
Gender							
Male	75 (37.88%)	27 (50.00%)	38 (74.51%)	<0.001	NS	S	S
Female	123 (62.12%)	27 (50.00%)	13 (25.49%)				
NYHA class							
I	2 (1.03%)	0 (0%)	0 (0%)	<0.001	NS	NoP	NoP
II	140 (70.71%)	32 (59.26%)	16 (31.37%)		NS	S	S
III	53 (26.77%)	19 (35.19%)	25 (49.02%)		NS	S	NS
IV	3 (1.52%)	3 (5.56%)	10 (19.61%)		NS	S	NS
Death							
No	186 (93.94%)	52 (96.30%)	43 (84.31%)	0.033	NS	NS	NS
Yes	12 (6.06%)	2 (3.70%)	8 (15.69%)				

Note: SD = standard deviation, Q1 = 25 percentage quartile, Q3 = 75 percentage quartile, NYHA = New York Heart Association, NoP = Comparison not performed because one of its column proportion is equal to zero or one.

**Table 2:** Co-morbidities by LVEF categories.

History of:	LVEF >50% (*n* = 198)	LVEF 49–40% (*n* = 54)	LVEF <40% (*n* = 51)	*p*-Value	*p*-Value 1	*p*-Value 2	*p*-Value 3
History of T2DM							
No	129 (65.15%)	33 (61.11%)	20 (39.22%)	0.003	NS	S	NS
Yes	69 (34.85%)	21 (38.89%)	31 (60.78%)				
H/of CAD							
No	103 (52.02%)	25 (46.30%)	20 (39.22%)	0.243	NS	NS	NS
Yes	95 (47.98%)	29 (53.70%)	31 (60.78%)				
H/of arrhythmias							
No	86 (43.43%)	22 (40.74%)	23 (45.10%)	0.899	NS	NS	NS
Yes	112 (56.57%)	32 (59.26%)	28 (54.90%)				

**Table 3:** Laboratory test results by LVEF categories.

	LVEF >50% (*n* = 198)	LVEF 49–40% (*n* = 54)	LVEF <40% (*n* = 51)	*p*-Value	*p*-Value 1	*p*-Value 2	*p*-Value 3
Na^+^ (mmol/L)							
Mean (SD)	139.35 (4.950)	139.54 (5.255)	138.92 (5.176)	0.808	NS	NS	NS
Min; Max	122; 152	116; 147	124; 148				
Median (Q1; Q3)	140.0 (138.0; 142.0)	141.0 (138.0; 143.0)	140.0 (137.0; 141.5)				
K^+^ (mmol/L)							
Mean (SD)	4.327 (0.7717)	4.463 (0.7088)	4.418 (0.8021)	0.027	NS	S	NS
Min; Max	1.7; 7.5	3.3; 6.2	2.7; 6.4				
Median (Q1; Q3)	4.40 (3.90; 4.80)	4.50 (3.90; 5.00)	4.60 (3.95; 5.25)				
Creatinine (mg/dL)							
Mean (SD)	1.5941 (0.75901)	1.6957 (0.61205)	1.9080 (0.95739)	0.035	NS	S	NS
Min; Max	0.52; 4.97	0.90; 3.50	0.71; 7.23				
Median (Q1; Q3)	1.420 (1.060; 1.860)	1.585 (1.230; 1.930)	1.710 (1.445; 2.170)				
eGFR (MDRD) (mL/min)							
Mean(SD)	46.535 (21.2707)	42.222 (16.7025)	42.143 (17.3025)	0.196	NS	NS	NS
Min; Max	8.3; 124.6	13.2; 87.2	8.3; 83.6				
Median (Q1; Q3)	43.90 (32.10; 57.70)	43.35 (28.30; 50.40)	39.90 (30.20; 51.40)				
Uric acid (mg/dL)							
Mean (SD)	7.328 (2.5407)	7.091 (2.0394)	7.798 (2.5859)	0.004	NS	S	S
Min; Max	1.7; 17.2	4.0; 12.8	5.1; 17.1				
Median (Q1; Q3)	7.05 (5.40; 8.60)	6.65 (5.40; 8.70)	8.20 (6.50; 9.90)				
NT-pro BNP							
Mean (SD)	2422.86 (1491.644)	3699.20 (1731.444)	6667.92 (2714.724)	<0.001	S	S	S
Min; Max	155; 10,209	1654; 9120	1884; 11,640				
Median (Q1; Q3)	1770 (1524; 3128)	3075 (2613; 4800)	6430 (4617; 9130)				
Left atrial volume							
Mean (SD)	62.41 (10.752)	80.85 (5.839)	102.39 (12.405)	<0.001	S	S	S
Min; Max	35; 91	69; 90	84; 130				
Median (Q1; Q3)	64.0 (53.0; 70.0)	81.0 (75.0; 87.0)	99.0 (93.0; 113.5)				
LDL							
Mean (SD)	111.14 (28.300)	137.04 (30.148)	146.78 (26.532)	<0.001	S	S	NS
Min; Max	50; 190	70; 190	76; 190				
Median (Q1; Q3)	110.0 (90.0; 130.0)	133.0 (114.0; 165.0)	150.0 (131.0; 168.0)				
HDL							
Mean (SD)	43.52 (6.705)	41.02 (6.257)	38.61 (6.422)	<0.001	S	S	NS
Min; Max	31; 60	30; 60	25; 52				
Median (Q1; Q3)	44.0 (38.0; 48.0)	40.0 (38.0; 45.0)	38.0 (33.0; 43.0)				
TG							
Mean (SD)	157.34 (52.341)	164.91 (58.606)	184.71 (59.069)	0.007	NS	S	NS
Min; Max	80; 304	90; 290	100; 308				
Median (Q1; Q3)	145.0 (112.0; 190.0)	154.0 (117.0; 200.0)	190.0 (133.0; 205.5)				

**Table 4:** FIB-4 score, LVEF and NT-pro BNP in patients with and without diabetes and hypertension.

Patients with Diabetes		FIB-4
LVEF	Correlation Coefficient (r)	0.378
	*p*-value	<0.001
NT-pro BNP	Correlation Coefficient (r)	0.045
	*p*-value	0.621
Patients without diabetes		FIB-4
LVEF	Correlation Coefficient (r)	0.344
	*p*-value	<0.001
NT-pro BNP	Correlation Coefficient (r)	0.001
	*p*-value	0.993
Patients with hypertension		FIB-4
LVEF	Correlation Coefficient (r)	0.355
	*p*-value	<0.001
NT-pro BNP	Correlation Coefficient (r)	0.002
	*p*-value	0.979
Patients without hypertension		FIB-4
LVEF	Correlation Coefficient (r)	0.506
	*p*-value	0.003
NT-pro BNP	Correlation Coefficient (r)	−0.100
	*p*-value	0.580

## Data Availability

The original contributions presented in the study are included in the article, and further inquiries can be directed to the corresponding author.
